# 

**Published:** 1854-06

**Authors:** 


					﻿No. I.]
JUNE.
[Vol. X-
BUFFALO
MEDICAL JOURNAL
IHouthh) Ikuiw
OF
MEDICAL AND SURGICAL SCIENCE.
*
AUSTIN FLINT, M. I).,	;
SANFQRD B. HUNT, M. Th. $ Er”T ;KK-
BUFFALO, N. Y.
L PUBLISHED BY JEWETT, THOMAfi A C <).
Commer«ial Advertiser Bui’dir.^.
1854.	.
Price 82.50 per anrr.m. in advance.
				

